# Causal relationship between gut microbiota and hepatocellular carcinoma: a two-sample Mendelian randomization and case–control study

**DOI:** 10.1007/s12672-025-02806-7

**Published:** 2025-06-03

**Authors:** Liu Yang, Wei Wei, Yuekui Wang, Wentao Kuai, Liang Xu

**Affiliations:** 1 Clinical School of The Second People’s Hospital Affiliated to Tianjin Medical University, Tianjin, 300192 China; 2 Tianjin Institute of Hepatology, Tianjin Second People’s Hospital, Tianjin, 300192 China

**Keywords:** Gut microbiota, Mendelian randomization, Case–control study, Hepatocellular carcinoma, Causal inference

## Abstract

**Background:**

Accumulating evidence from both observational studies and clinical trials has established a connection between the gut microbiome and hepatocellular carcinoma (HCC). Nevertheless, the causal relationship between gut microbes and hepatocellular carcinoma remains ambiguous.

**Methods:**

The principal study method employed was Mendelian randomization (MR). The exposure group included 7738 samples from the MiBioGen Consortium Gut Microbiota Genome-Wide Association Study (GWAS), whereas the outcome group included 8,885,115 single nucleotide polymorphisms (SNPs) from the IEU OpenGWAS project database of hepatocellular carcinoma (HCC). Utilizing the MR-Egger regression, weighted median (WME), and weighted mode (WM) as supplemental methods for inverse variance weighting (IVW), the main basis was IVW. Additional sensitivity, pleiotropy, and heterogeneity tests were performed. To confirm the MR results, sequencing data from a case–control study were used. We enrolled 29 patients with HBV-HCC who were hospitalized at Tianjin Second People's Hospital between October 2022 and August 2023, and chose 21 healthy employees as controls.

**Results:**

IVW analysis showed that gut Clostridia (OR = 0.554, 95%CI: 0.361–0.850, *p* = 0.007), Clostridiales (OR = 0.554, 95%CI: 0.361–0.850, *p* = 0.007), and Dorea (OR = 0.679, 95%CI: 0.479–0.964, *p* = 0.030) were protective factors against HCC. Gut Desulfovibrio piger (OR = 1.304, 95%CI: 1.080–1.499, *p* = 0.004), Bacteroides ovatus (OR = 1.304, 95%CI: 1.046–1.626, *p* = 0.018), Bacteroides stercoris (OR = 1.714, 95%CI: 1.183–2.483, *p* = 0.004), and Paraprevotella xylaniphila (OR = 1.256, 95%CI: 1.003–1.573, *p* = 0.047) were risk factors for HCC. A case–control study demonstrated that the relative abundances of gut Clostridia, Clostridiales, and Dorea were significantly higher in healthy controls than in patients with HCC. Conversely, the relative abundance of Bacteroides stercoris was lower in healthy controls than in patients with HCC. Conclusion: This study showed that gut Clostridia, Clostridiales, and Dorea were associated with a reduced risk of HCC, whereas gut Bacteroides stercoris was the exact opposite.

**Conclusion:**

This study demonstrated that gut Clostridia, Clostridiales, and Dorea were linked to a decreased HCC risk, while gut Bacteroides stercoris was linked to an increased HCC risk.

## Introduction

Hepatocellular carcinoma (HCC), a pathological form of liver cancer, constitutes a significant portion of liver cancer cases in terms of both incidence and mortality [1–3]. The absence of specific symptoms often leads to late diagnosis in most patients, highlighting the pressing need to identify underlying causes and implement effective preventive measures to alleviate the disease burden.

Gut microbiota is a group of bacteria that are designated to reside in the human gut and depend on the human body for a long time, an indispensable part of the human body [4]. They not only involved in various physiological processes such as digestion, metabolic regulation and immune system function, but also affect the body's immune system regulation, digestion, metabolism, epithelial cell proliferation, insulin resistance, and even behavioral and neurological functions [5, 6].

Disturbance of the gut microbiota has adverse effects on human health and is a major cause of various chronic diseases [7]. An imbalance in bacterial homeostasis may promote HCC through multiple mechanisms when the diversity of the intestinal flora is reduced, pathogenic bacteria are overpopulated, or probiotics are depleted. Emerging evidence suggests that the gut microbiota is intricately linked to the onset and progression of hepatocellular carcinoma [8, 9]. Its associated metabolites and components can modulate the activation of multiple signaling pathways, thereby significantly influencing the development of hepatocellular carcinoma [10]. For example, Lau et al.[11] found that Lactobacillus acidophilus can inhibit the Rho-GTPase signaling pathway by metabolizing valeric acid, thus inhibiting HCC progression. In addition, it has been observed that acetate produced by Bifidobacterium pseudonidarum can inhibit the oncogenic IL-6/JAK1/STAT3 signaling pathway, thereby significantly suppressing the progression of HCC [12]. Furthermore, Gram-negative bacteria can activate the hepatic pattern recognition receptor Toll-like receptor 4 (TLR4), thereby promoting HCC development through the nuclear factor κB (NF-κB) signaling pathway [13]. Some researchers have proposed that an imbalance in the intestinal flora can promote the development of HCC by Reconstructing the inflammatory microenvironment [14]. In a clinical trial of HCC patients, orally administering a probiotic bacteria cocktail improved overall 1-year survival [15]. The gut microbiota has shown the possibility of being a potential therapeutic target and biomarker in the occurrence and development of HCC.

Some studies have shown that local combined systemic therapy can significantly improve the 5-year survival rate of patients with advanced HCC with macrovascular invasion [16]. Li Y. K. et al. showed in a retrospective study that novel imaging biomarkers can predict early recurrence of HCC [17]. Some previous meta-analyses have pointed out that tumor-associated lymphatic vessel density (LVD) as a new biomarker can drive early stratification of high-risk patients with hepatobiliary cancer [18, 19]. These breakthrough findings highlight the critical importance of identifying novel biomarkers and therapeutic targets to achieve early warning and precise intervention of HCC.

Mendelian randomization (MR) is a commonly used statistical method in epidemiology and is now widely used to study various diseases. Single nucleotide polymorphisms (SNPs) are variations in DNA sequences that arise from alterations in individual nucleotides at the genomic level [20, 21] and are advantageous in that they are not influenced by acquired confounding factors. In contrast to the randomized exposure assignment used in traditional observational studies or randomized controlled trials (RCTs) [22, 23], MR provides several distinct advantages. This eliminates the problem of reverse causation and reduces the number of confounding variables less of a factor. Additionally, there are significant benefits in terms of ethical considerations and practicality. The 16S rRNA amplicon sequencing technique analyzes the microbial community structures in environmental samples by targeting specific variable regions, amplifying them by PCR using universal primers for conserved areas, and sequencing these highly variable segments. This method is essential for studying the composition of the microbial communities in the environment. Considering these advantages, we employed two-sample MR using 16S rRNA sequencing technology in this study. By investigating the role of the gut microbiota in HCC, we can enhance our understanding of the pathogenesis of HCC and uncover potential new insights and strategies for its prevention and treatment.

## Materials and methods

### Three assumptions of MR

The primary aim of this study was to explore the causal relationship between gut microbiota and HCC. The MR analysis is underpinned by three fundamental assumptions. Firstly, it presumes that the IVs employed in the analysis are truly correlated with the exposure factor under investigation. Secondly, it is assumed that the IVs are independent of any possible confounding factors, guaranteeing that the detected associations are not swayed by external variables. Finally, the analysis supposes that the effects of the IVs on outcomes are mediated solely via the exposure factor, thereby offering a more lucid comprehension of the underlying mechanisms at work.

### Data sources

We compiled data from a Netherlands genome-wide association study (GWAS) of the intestinal microbiome. In total, there were 207 microbial taxa in this collection, representing five phyla, 10 classes, 13 orders, 26 families, 48 genera, and 105 species. Additionally, 205 functional pathways were identified. Among the 197,611 subjects included in the IEU OpenGWAS project's HCC dataset were 1866 patients with HCC and 195,745 healthy controls; the study involved 8,885,155 SNPs. Ethical approval was obtained from the appropriate review boards for the original investigations that used these data sources. No further ethical clearance was necessary for this investigation because it was built upon an authorized research strategy.

### IVs selection

The selection criteria outlined below were used to identify the IVs: (1) SNPs exhibiting a threshold of significance (*p* < 1.0 × 10–5) within the range of gene loci were identified and utilized as potential IVs; (2) SNPs with linkage disequilibrium (r2 < 0.001) were excluded; (3) the width of the region was established at 10,000 kb to guarantee the independence of each SNP; and (4) SNPs with an F-statistic greater than 10 were selected to exclude weak IVs. Three fundamental assumptions should be met by the IVs after screening: first, that the IVs and exposure factors are strongly correlated; second, that the IVs are not associated with other outcome variables that could confound the results; and third, that the IVs exclusively affect the outcomes via exposure rather than through other means. The final selection for instrumental variables was based on the screening criteria, which led to the retention of 45 SNPs linked to known bacterial genera.

### MR analysis

Four popular MR Methods were used: inverse variance weighting (IVW), MR-Egger regression, weighted median (WME), and weighted mode (WM). Tests for heterogeneity, pleiotropy, and sensitivity were also performed. The IVW method served as the key analysis technique, whereas the other three approaches served as supplemental techniques to enhance the trustworthiness of the results. A positive outcome was initially deemed when IVW was less than 0.05. There was no variability among the SNPs related to each flora unit, as determined by Cochran's Q test (Q > 0.05). MR-Egger regression was employed to determine whether the genetic instrument exhibited horizontal pleiotropy on the outcome. (*p* > 0.05), indicating the absence of horizontal pleiotropy. The intercept term in the MR-Egger regression model shows the average pleiotropy of the instrument variable. Leave-one-out test was used to investigate the sensitivity of a single SNP. After removing one SNP, the results mostly remained unchanged, suggesting that they were reliable. All studies using Mendelian randomization were performed in the R statistical software environment, specifically utilizing the TwoSampleMR package (version 4.4.0).

### Case–control study

From October 2022 through August 2023, researchers from Tianjin Second People's Hospital conducted a case–control study. We recruited 21 healthy controls and 35 patients diagnosed with HCC. Inclusion of patients with a history of chronic hepatitis B infection and postoperative tumor biopsy confirmation of HBV-HCC. Patients with severe gastrointestinal disease or other tumors, recent antibiotic or medication use, inability to provide adequate stool samples, or inadequate clinical information were not considered. All healthy controls and patients with HCC had their stool samples collected before treatment. A total of 29 patients with HCC and 21 healthy controls were included. All study participants provided basic demographic and laboratory information. This includes factors such as gender, age, etiology, BCLC stage, alanine aminotransferase (ALT), total bilirubin (TBIL), albumin (ALB), alpha-fetoprotein (AFP) level, and Child–Pugh. This study protocol was approved by the Tthe Medical Ethics Committee of Tianjin Second People's Hospital (Approval No. Jin Er Ren Min Lun Shen Zi [2024] No. 39) and all participants signed an informed consent form. The procedures used in this study adhere to the tenets of the Declaration of Helsinki.

#### Storage of specimens and 16 s rRNA Sequencing

Each participant's sample was promptly placed in liquid nitrogen and stored at − 80 °C after collection. DNA content and purity were determined using 1% agarose gel electrophoresis. This concentration was used to prepare a diluted DNA solution at a concentration of 1 ng/µL. ITS, 16S rRNA, and 18S rRNA genes were amplified from separate areas using specific primers with barcodes. The PCR products were combined with an equal volume of loading buffer containing SYBR Green before electrophoresis on 2% agarose gel for detection. Equimolar ratios of the PCR products were then mixed. A gel extraction kit (Qiagen) was used to purify the PCR products. A TruSeq® DNA PCR-Free Sample Preparation Kit was used to incorporate index codes during the preparation of the sequencing library. And then indexing codes were integrated. To evaluate library quality, both a Thermo Scientific Qubit@ 2.0 fluorometer and an Agilent Bioanalyzer 2100 system were utilized. Finally, libraries were sequenced on an Illumina NovaSeq platform.

#### Sequencing data analysis

For data normality evaluation, we used the Shapiro–Wilk test; for comparing the relative abundance of gut microbiota between the HCC and healthy control groups, we used the Mann–Whitney U test. The data analysis was conducted using SPSS 27.0. The statistical significance of the results was defined as p < 0.05. Plotting was performed using GraphPad Prism, version 10.

## Results

### Mendelian randomization

#### IVs selection

According to the screening criteria, 45 SNPs associated with known bacterial genera were retained as instrumental variables (*p* < 1.0 × 10–5, r2 < 0.001). Six SNPs were linked to Clostridia at the class level, 6 SNPs to Clostridiales at the order level, 7 SNPs to Dorea at the genus level, and 5 SNPs to Clostridia at the class level. Bacteroides ovatus, Bacteroides stercoris, Desulfovibrio piger, and Bacteroides stercoris were linked to 5, 10, 5, and 6 SNPs, respectively, at the species level. All IVs had F-statistics greater than 10 to eliminate weak instrumental variables.

#### Causal inference

**Fig. 1:**
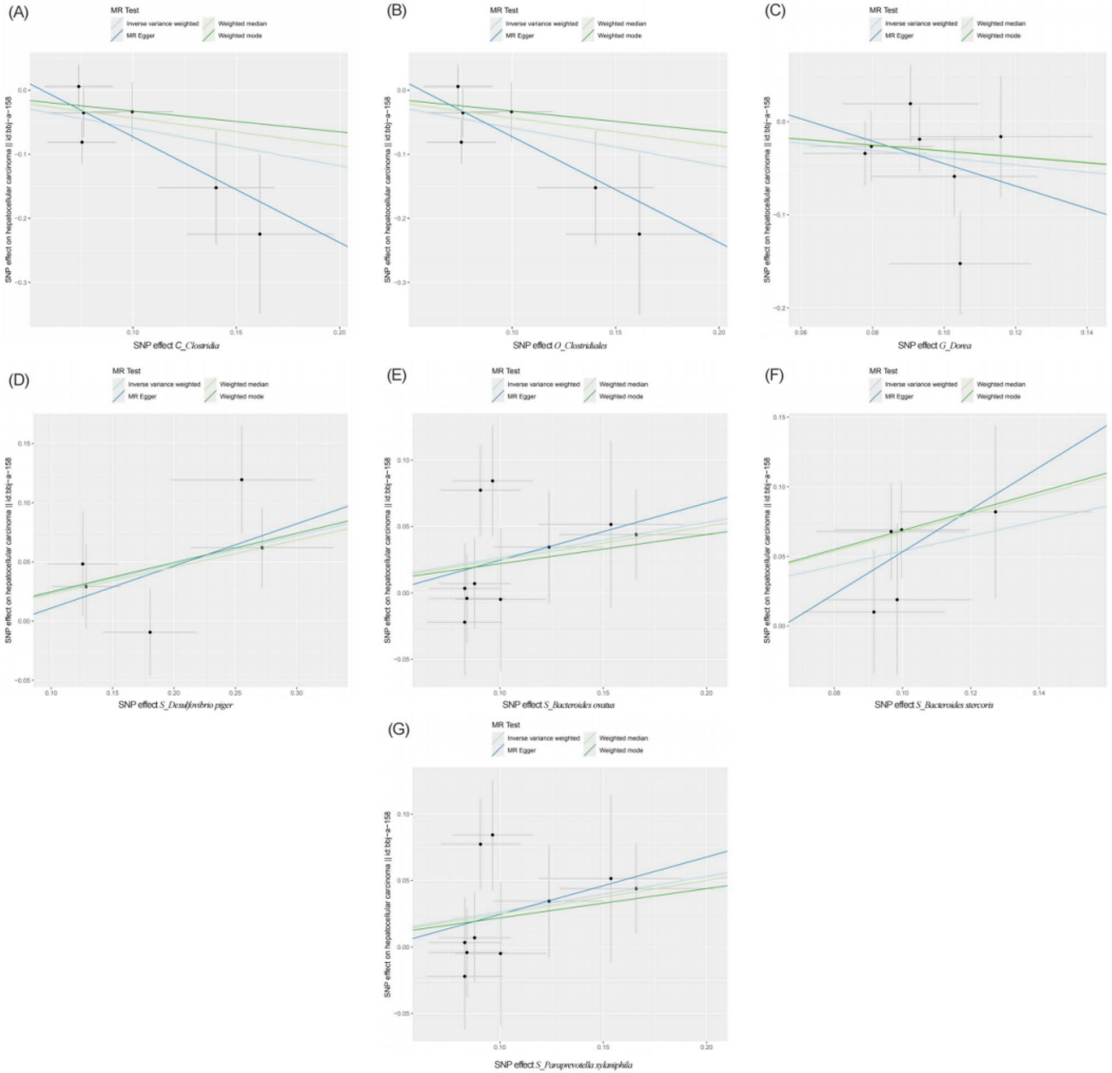
Scatter plots showing causal relationship between exposure and outcome

To determine whether exposure had any effect on the outcome, we primarily used the IVW approach. To ensure that there was no bias, we also employed MR-Egger regression, WME, and WM as additional methods. Scatter plots were created (Fig. 1). Seven microbiota abundances were found to be significantly associated with HCC after utilizing IVW to evaluate particular relationships (*p* < 0.05) (Table 1). According to IVW analysis, gut Clostridia (OR = 0.554, 95%CI: 0.361–0.850, *p* = 0.007), Clostridiales (OR = 0.554, 95%CI: 0.361–0.850, *p* = 0.007), and Dorea (OR = 0.679; 95%CI: 0.479–0.964, *p* = 0.030) were protective factors against HCC. Desulfovibrio piger (OR = 1.304, 95%CI: 1.080–1.499, *p* = 0.004), Bacteroides ovatus (OR = 1.304, 95%CI: 1.046–1.626, *p* = 0.018), Bacteroides stercoris (OR = 1.714, 95%CI: 1.183–2.483, *p* = 0.004), and Paraprevotella xylaniphila (OR = 1.256, 95%CI: 1.003–1.573, *p* = 0.047) were risk factors for HCC.

**Table 1:** MR estimates for the association between gut microbiota and HCC

Level	Bacterial taxa	MR method	nSNP	OR	95%CI	*p*-value
Class	Clostridia	IVW	6	0.554	0.361–0.850	0.007
		MR-Egger	6	0.192	0.029–1.283	0.164
		Weighted median	6	0.649	0.360–1.170	0.150
		Weighted mode	6	0.722	0.312–1.672	0.482
Order	Clostridiales	IVW	6	0.554	0.361–0.850	0.007
		MR-Egger	6	0.192	0.029–1.282	0.164
		Weighted median	6	0.649	0.366–1.151	0.139
		Weighted mode	6	0.723	0.327–1.596	0.458
Genus	Dorea	IVW	7	0.679	0.479–0.964	0.030
		MR-Egger	7	0.300	0.015–6.132	0.470
		Weighted median	7	0.733	0.469–1.146	0.173
		Weighted mode	7	0.729	0.407–1.307	0.329
Species	*Desulfovibrio piger*	IVW	5	1.272	1.080–1.499	0.004
		MR-Egger	5	1.428	0.776–2.627	0.335
		Weighted median	5	1.256	1.000–1.578	0.050
		Weighted mode	5	1.280	0.984–1.666	0.140
Species	*Bacteroides ovatus*	IVW	10	1.304	1.046–1.626	0.018
		MR-Egger	10	1.539	0.679–3.489	0.332
		Weighted median	10	1.285	0.939–1.758	0.118
		Weighted mode	10	1.246	0.866–1.793	0.267
Species	*Bacteroides stercoris*	IVW	5	1.714	1.183–2.483	0.004
		MR-Egger	5	4.561	0.084–2.724	0.510
		Weighted median	5	1.956	1.224–3.126	0.005
		Weighted mode	5	1.988	1.101–3.589	0.085
Species	*Paraprevotella xylaniphila*	IVW	6	1.256	1.003–1.573	0.047
		MR-Egger	6	0.841	0.138–5.136	0.860
		Weighted median	6	1.232	0.921–1.647	0.160
		Weighted mode	6	1.234	0.872–1.748	0.289

#### Sensitivity analyses

**Fig. 2:**
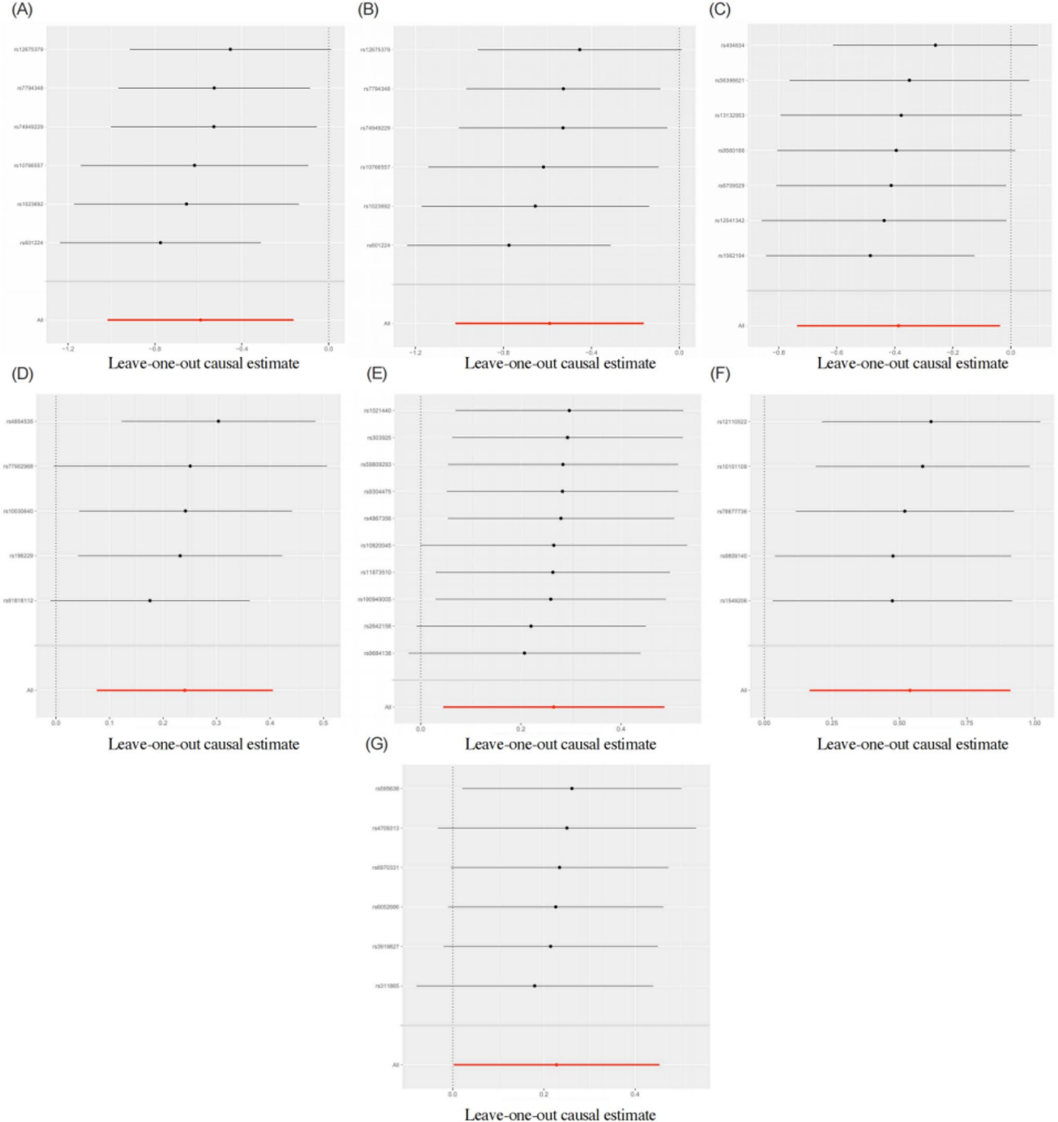
Leave-one-out plots analysis revealed no outlying SNPs

The SNPs strongly linked to the seven aforementioned gut microorganisms did not exhibit any statistical heterogeneity according to the Cochran Q test (*p* > 0.05). Table 2 shows that SNPs strongly linked to the aforementioned seven gut bacteria did not exhibit horizontal pleiotropy (*p* > 0.05) when MR-Egger regression intercept term analysis was performed. Figure 2 shows that there were no outlying instrumental factors because the results of MR Analysis remained unchanged after eliminating a single SNP, according to leave-one-out analysis.

**Table 2:** Cochran Q test and MR-Egger regression analysis showed that SNPs were highly correlated with intestinal flora

No.	Level	Bacterial taxa	IVW		MR-Egger	
			Q	*p*-value	Intercept	*p*-value
1	Class	Clostridia	5.472	0.361	0.093	0.325
2	Order	Clostridiales	5.471	0.361	0.093	0.324
3	Genus	Dorea	6.627	0.357	0.0075	0.616
4	Species	*Desulfovibrio piger*	3.990	0.407	− 0.025	0.723
5	Species	*Bacteroides ovatus*	7.274	0.609	− 0.019	0.691
6	Species	*Bacteroides stercoris*	1.644	0.801	− 0.098	0.662
7	Species	*Paraprevotella xylaniphila*	1.170	0.948	0.064	0.684

### Case-control study

Fifty people participated in the case-control study: 21 were healthy controls and 29 were HCC patients. Table 3 displays the demographic and laboratory data from the beginning of the study for both groups as well as comparisons between them.

**Table 3:** Baseline demographics and laboratory data between the healthy controls and HCC patients

Variables	Healthy controls (n = 21)	HCC patients (n = 29)	t/x^2^	*p*-value
Gender			1.900	0.168
Male	7(33.3%)	25(86.2%)		
Female	14(66.7%)	4(13.8%)		
Age (years)	49.40(42.00,56.00)	56.55(49.00,61.00)	0.853	0.425
Etiological component			–	–
HBV-related	NA	29(100%)		
Other	NA	0(0%)		
BCLC stage			–	–
A/B	NA	18(62.07%)		
C/D	NA	11(37.93%)		
ALT (U/L)			–	–
≤ 50	21 (100%)	23(79.3%)		
> 50	0(0%)	6(20.7%)		
TBIL (μmol/L)			0.702	0.402
≤ 20	20(95.2%)	13(44.8%)		
> 20	1(4.8%)	16(55.2%)		
ALB (g/L)			0.861	0.353
≤ 40	1(4.8%)	16(55.2%)		
> 40	20(95.2%)	13(44.8%)		
AFP level (ng/ml)			–	–
≤ 400	NA	23(79.31%)		
> 400	NA	6(20.69%)		
Child-Pugh			–	–
A	NA	21(72.4%)		
B	NA	8(27.6%)		

#### Analysis of diversity

**Fig. 3:**
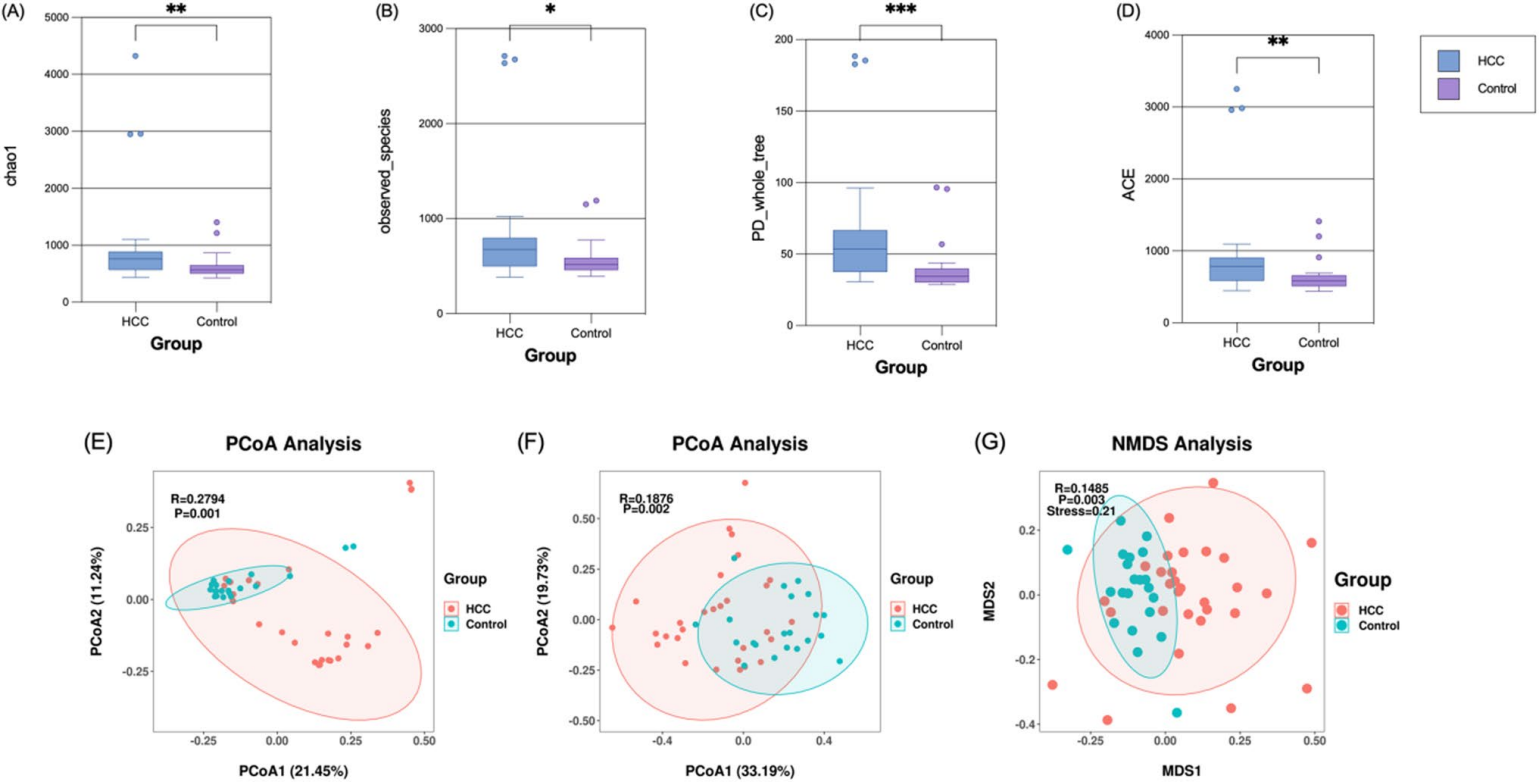
Dissimilarities in the make-up and distribution of the gut microbiota between individuals with HCC and healthy controls. The measures used to characterize α-diversity were the Chao1 index (**A**), the observed species (**B**), the PD index (**C**), and the ACE index (**D**). PCoA, which is based on the Unweighted UniFrac distance measure (**E**) and the Weighted UniFrac distance metric (**F**), was used to describe β-diversity. Together with the prior analytical methodologies, Non-Metric Multi-Dimensional Scaling (NMDS) analysis (G) was employed. *p < 0.05, **p < 0.01, ***p < 0.001

α-Diversity and β-diversity serve as crucial indicators for characterizing the microbial composition and distribution. α-Diversity was used to assess the diversity of the microbial community within a sample [24]. Analysis of diversity in a single sample reflects both the richness and variability of the microbial community. β-Diversity is a comparative analysis of microbial community composition in different samples. We selected Chao1, observed species, PD, and ACE indices as further descriptions of α-diversity. The boxplot of the Chao1 index showed that the difference between healthy controls and patients with HCC was statistically significant (*p* = 0.0086) (Fig. 3A). The box plot of observed species showed that the intergroup comparison between healthy controls and HCC patients was statistically significant (*p* = 0.0341) (Fig. 3B). The PD index showed that HCC patients were statistically significant compared with healthy controls (*p* = 0.0006) (Fig. 3C). The ACE index showed a statistically significant difference between patients with HCC and healthy controls (*p* = 0.0091) (Fig. 3D). We performed PCoA and Non-metric multi-dimensional scaling (NMDS) analyses based on the Unweighted Unifrac distance and Weighted Unifrac distance for β-diversity assessment. PCoA analysis showed that, PCoA analysis based on Unweighted Unifrac distance (R = 0.2794, *p* = 0.001) and Weighted Unifrac distance PCoA analysis (R = 0.1876, *p* = 0.002) showed statistically significant gut microbiota composition in both groups (Fig. 3E, 4F). NMDS analysis (R = 0.1485, stress = 0.21, *p* = 0.003) yielded consistent conclusions (Fig. 3G).

**Fig. 4:**
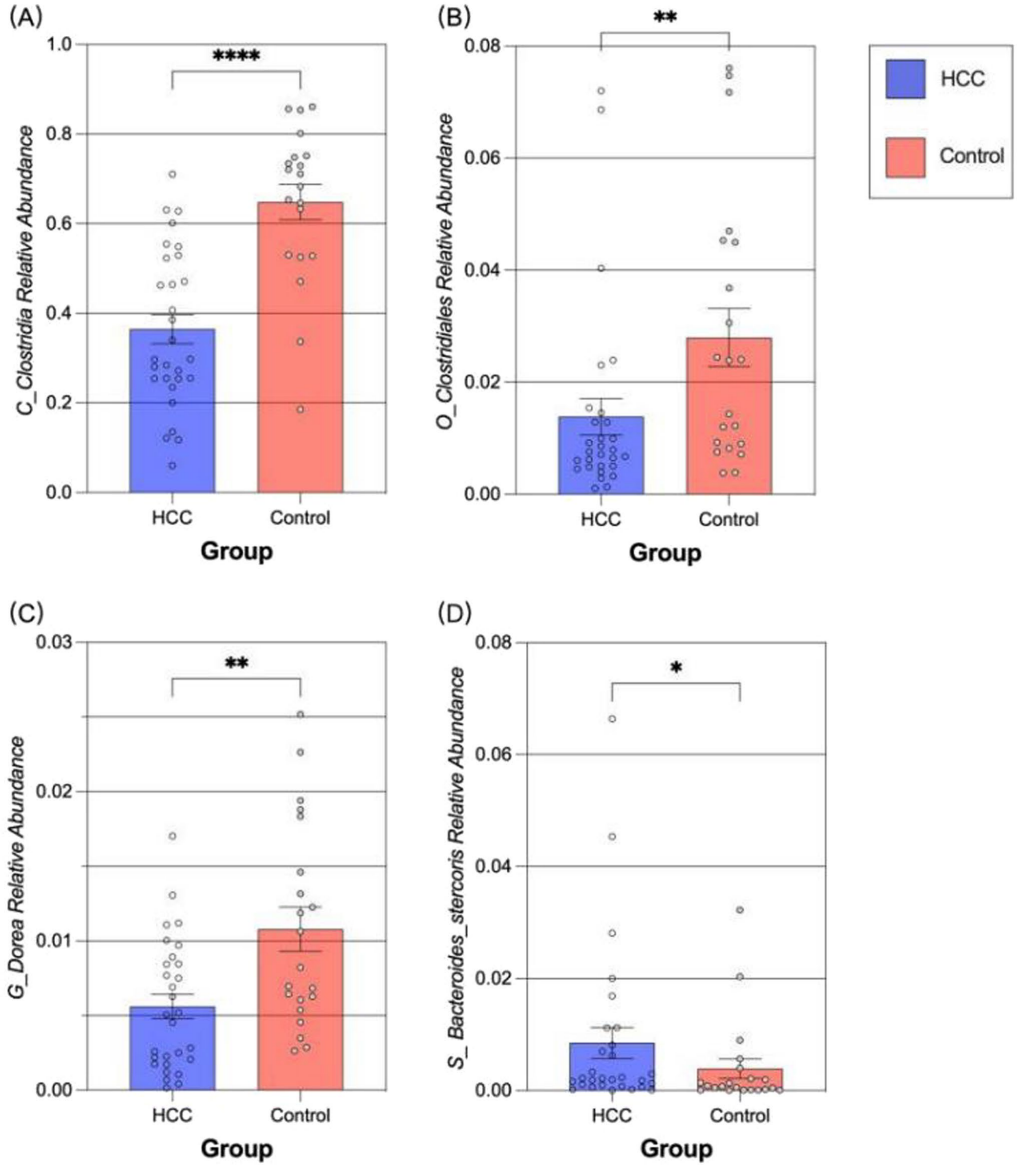
Bar graph of validation MR. Comparison of the relative abundance of Class_Clostridia (**A**), Order_Clostridiales (**B**), Genus_Dorea (**C**) as well as Species_Bacteroides_stercoris (**D**) in healthy controls and HCC patients. *P < 0.05, **P < 0.01, ****P < 0.0001

#### Validation of MR results

Mendelian randomization was used to establish causation, which was subsequently confirmed using data from the case–control studies. For this bar graph, we isolated and compared the relative abundance of Clostridia, Clostridiales, Dorea, and Bacteroides stercoris in healthy controls and patients with HCC. Figure 4A–C show that compared to patients with HCC, healthy controls had a higher relative abundance of Clostridia (*p* < 0.0001), Clostridiales (*p* < 0.01), and Dorea (*p* < 0.01). The relative abundance of Bacteroides stercoris was lower in the healthy control group than in the HCC group (*p* < 0.05) (Fig. 4D). Owing to the lack of species-level annotation in many of the 16S sequencing sequences, we were unable to determine whether Desulfovibrio piger, Bacteroides ovatus, and Paraprevotella xylaniphila were more abundant in one group than in the other.

## Discussion

An increasing number of studies in recent years have linked changes in gut flora to cancer and other chronic diseases. This confirms that gut flora has a significant impact on human health [25]. Because of the tight link between gut flora and the liver in gut-liver circulation, intestinal flora or its metabolites can influence liver disorders under direct physiological settings [26–28].

In this study, the causal association between intestinal flora and hepatocellular carcinoma (HCC) was revealed for the first time at the species level using a combination of MR and case–control studies. Our study differs from previous MR studies in that it used HCC as the only outcome variable. We utilized the broader Dutch Microbiome Project (DMP) [29], which includes 207 microbial taxa and 205 functional pathways. Enabling the discovery of more intestinal flora relevant to HCC. The results showed that Clostridia, Clostridiales, and Dorea were protective flora against HCC, whereas Bacteroides stercoris and Desulfovibrio piger were risk factors. This case–control study further validated the enrichment of Clostridia, Clostridiales, and Dorea in healthy populations, which is highly consistent with the MR results. Compared with previous studies, this study established the flora risk profile of HCC at the species level, providing a finer target for the study of the mechanism of intestinal flora regulation of HCC, breaking through the limitations of previous associations at the family or genus level. For example, Ma et al. [30] only reported the protective effect of intestinal flora on HCC at the family level. In the sequencing results, we did not find relative abundances of Vibrio desulfuriae, Bacteroides faecalis, and Pseudomonas xylomonas. This was because a large portion of the 16S sequencing sequences was not annotated at the species level, which prevented us from drawing any conclusions about the differences between the two groups. Similarly, the relative abundance of the associated gut microbiome was higher in the HCC group, indicating that these microbiomes increased disease risk, and it was higher in healthy controls, suggesting that these microbiomes demonstrated protective properties during health. Wang et al. [31] found that Dorea had a protective effect on the occurrence of cholangiocarcinoma through MR Research, which may affect the immune microenvironment of the liver and promote or inhibit inflammation. And changes in the immune response of the liver may promote or protect the development of cancer cells [32]. Our findings are consistent with this, showing that Dorea has a potential cross-cancer protective effect.

From the perspective of clinical translation, This research offers novel concepts for the prevention and treatment of HCC: The results of recently conducted clinical trials have shown that the combined use of probiotics and antiviral therapy can significantly improve intestinal dysbiosis in patients with HBV-associated cirrhosis, which in turn has a delaying effect on the progression of HCC [29]. Therefore, with the help of supplementation with probiotics associated with Clostridia or Dorea as well as dietary fiber, it may be expected to reduce the risk of liver cancer. Second, selective transplantation of fecal microorganisms rich in protective flora has the potential to be a novel preventive tool for high-risk populations such as cirrhotic patients. The flora identified in this study, such as Bacteroides stercoris, can be used as a secondary marker for early screening of HCC. With the help of longitudinal tracking of changes in gut flora in cirrhotic patients, such as the persistent elevation of Desulfovibrio piger, it may be possible to predict the risk of transformation of HCC, and thus provide guidance on the timing of clinical interventions.

This study has certain limitations: (1) Three levels of gut microbiota have not been demonstrated in case–control studies due to the limitations of 16 s RNA sequencing. (2) All data in this study were obtained from a European population, and further studies are needed to confirm these results in the Asian population. In future case–control studies, we could use metagene sequencing, which has a greater depth of species identification and can fully validate our findings. (3) The small number of IVs in MR analysis may increase the risk of weak instrumental bias, and statistical validity may be improved in the future by joint analysis across databases.

In conclusion, we found that gut Clostridia, Clostridiales, and Dorea are protective factors against HCC, and gut Bacteroides stercoris is a risk factor for HCC. The findings of this study serve as a valuable reference for further investigation of the specific mechanisms through which intestinal flora influence the development of HCC.

## Data Availability

All data and materials supporting the findings of this work are available from the corresponding author upon reasonable request.
